# Mechanics Regulates Fate Decisions of Human Embryonic Stem Cells

**DOI:** 10.1371/journal.pone.0037178

**Published:** 2012-05-16

**Authors:** Yubing Sun, Luis G. Villa-Diaz, Raymond H. W. Lam, Weiqiang Chen, Paul H. Krebsbach, Jianping Fu

**Affiliations:** 1 Integrated Biosystems and Biomechanics Laboratory, University of Michigan, Ann Arbor, Michigan, United States of America; 2 Department of Mechanical Engineering, University of Michigan, Ann Arbor, Michigan, United States of America; 3 Department of Biologic and Materials Sciences, University of Michigan, Ann Arbor, Michigan, United States of America; 4 Department of Biomedical Engineering, University of Michigan, Ann Arbor, Michigan, United States of America; University of Newcastle upon Tyne, United Kingdom

## Abstract

Research on human embryonic stem cells (hESCs) has attracted much attention given their great potential for tissue regenerative therapy and fundamental developmental biology studies. Yet, there is still limited understanding of how mechanical signals in the local cellular microenvironment of hESCs regulate their fate decisions. Here, we applied a microfabricated micromechanical platform to investigate the mechanoresponsive behaviors of hESCs. We demonstrated that hESCs are mechanosensitive, and they could increase their cytoskeleton contractility with matrix rigidity. Furthermore, rigid substrates supported maintenance of pluripotency of hESCs. Matrix mechanics-mediated cytoskeleton contractility might be functionally correlated with E-cadherin expressions in cell-cell contacts and thus involved in fate decisions of hESCs. Our results highlighted the important functional link between matrix rigidity, cellular mechanics, and pluripotency of hESCs and provided a novel approach to characterize and understand mechanotransduction and its involvement in hESC function.

## Introduction

Much effort has been recently directed to investigate how soluble factors in the local cellular microenvironment of embryonic stem cells (ESCs) regulate their fate decisions [1], [2]; however, the effects of mechanical signals in the local cellular microenvironment on the fate of ESCs are still not yet well understood. Experimental evidence established in recent years has shown that mechanical signals experienced by ESCs through their biophysical interactions with the extracellular matrix (ECM) can play critical roles in regulating survival, proliferation and differentiation. For example, Saha *et al.* reported that cyclic mechanical stretches inhibit differentiation of human ESCs (hESCs) through the TGF-β/Activin/Nodal signaling pathway [3], [4]. Chowdhury *et al.* recently demonstrated that a local cyclic stress applied through focal adhesions (FAs) to mouse ESCs (mESCs) induce their spreading and differentiation [5]. In addition to external mechanical forces, matrix mechanics has also been shown to regulate lineage commitments of human mesenchymal stem cells [6], [7]. More recently, two independent studies have indicated that mESCs can sense and respond to subtle changes in matrix mechanics. First, Evans *et al.* [8] demonstrated that mESCs showed increased spreading, proliferation and osteogenic differentiation when plated on rigid poly-dimethylsiloxane (PDMS) substrates as compared to the cells plated on soft ones. The second study by Chowdhury *et al.* demonstrated that mESCs could maintain their pluripotency for a long term on soft polyacrylamide gels even without leukemia inhibitory factor (LIF), which is essential for maintaining pluripotency of mESCs [9]. hESCs are intrinsically different from mESCs, in regard to the required growth factors and dominant signal pathways that regulate their pluripotency [10]. For example, it has been suggested that activation of the bFGF/MAPK pathway is required for self-renewal of hESCs, yet inhibiting this pathway is known to promote self-renewal of mESCs [11], [12]. Collectively, there is still limited knowledge of how mechanical signals in the local cellular microenvironment regulate fate decisions of hESCs, and advancing in such knowledge will be critical for both fundamental understanding and clinical applications of hESCs. Therefore, this work was set to investigate explicitly the mechanosensitive properties of hESCs.

Recently, our group and others have proposed the use of microfabricated elastomeric PDMS micropost arrays to regulate substrate rigidity, independently of effects on adhesive and other material surface properties [7], [13], [14]. Our approach involves a library of replica-molded arrays of hexagonally spaced PDMS microposts from microfabricated silicon masters, which present the same surface geometry but different post heights, to control the substrate rigidity. The spring constant *K* of the PDMS micropost is solely determined by its geometry and by the *Young*'s modulus *E* of PDMS, and *K* can be approximately calculated using the *Euler-Bernoulli* beam theory as *K* = 3*πED^4^*/(64*L* ^3^), where *D* and *L* are the PDMS post diameter and height, respectively. The substrate rigidity of the PDMS micropost array can be further characterized using an effective *Young*'s modulus *E_eff_* of a continuous elastic substrate, and it is calculated using the expression of *E_eff_* = 9*K*/(2*πD*) [14]. Thus, the rigidity of the PDMS micropost array can be modulated by varying the post height *L* while keeping all other aspects of the substrate such as surface chemistry, ligand density, and bulk and nanoscale mechanics of PDMS unchanged.

In previous studies, it has been shown that rigidity of the PDMS micropost array can significantly impact cell morphology, FA formation, cytoskeleton contractility, and adult stem cell differentiation. The PDMS micropost array is also ideal for studies of involvement of cytoskeleton contractility in mechanoresponsive cellular behaviors, as the PDMS microposts can serve simultaneously as force sensors to map live-cell subcellular distributions of traction forces [7], [13]. In this study, we proposed to apply the PDMS micropost array to study the mechanosensitivity of hESCs and how matrix mechanics could regulate pluripotency of hESCs.

## Results and Discussion

Before plating hESCs on the PDMS micropost array, we first used microcontact printing to coat the tops of the PDMS microposts with vitronectin, which has been proved supportive for self-renewal of hESCs [15]. hESCs were further cultured in a chemically defined serum-free medium to establish a fully defined culture system [16], [17]. For all experiments two hESC lines, H1 and H9, were used with similar results.

**Figure 1:**
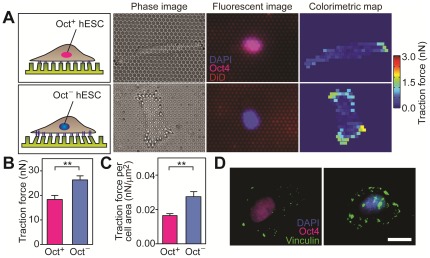
Differential cytoskeleton contractility and FA distribution for single Oct^+^ and Oct^−^ hESCs. (*A*) Quantification of subcellular traction forces for single Oct^+^ (top row) and Oct^−^ (bottom row) hESCs using the PDMS micropost array. (*B&C*) Bar plots of total traction forces per cell (*B*) and traction force per cell area (*C*) for both single Oct^+^ and Oct^−^ hESCs. Data represents the means ± s.e.m from 3 independent experiments. **, *p*<0.01. (*D*) Immunofluorescence images showing FA distributions in single hESCs (left: Oct^+^; right: Oct^−^), as indicated by vinculin staining. Scale bar, 20 µm.

**Figure 2:**
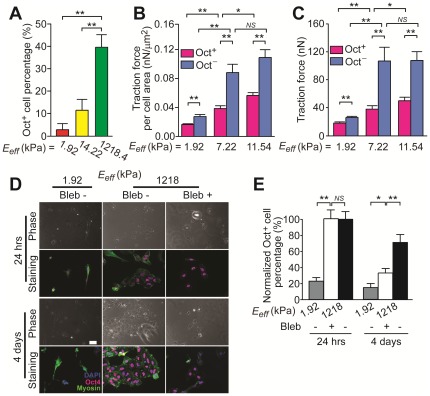
Matrix mechanic-mediated behaviors of single hESCs on PDMS micropost arrays with different rigidities. (*A*) Bar plot of percentage of Oct^+^ cells for single hESCs plated on the PDMS micropost arrays with different rigidities. (*B&C*) Traction force per cell area (*B*) and total traction forces per cell (*C*) for both Oct^+^ and Oct^−^ cells as a function of the PDMS micropost array rigidity. (*D*) Phase contrast and immunofluorescence images of hESCs treated with or without blebbistatin on both soft (*E_eff_* = 1.92 kPa) and rigid (*E_eff_* = 1,218.4 kPa) PDMS micropost arrays. Scale bar, 50 µm. (*E*) Bar plot of percentage of Oct^+^ cells for blebbistatin treated hESCs and untreated controls as a function of the PDMS micropost array rigidity. Data in *E* was normalized to the value for untreated hESCs plated on the rigid micropost array under the 24-hr treatment condition. Data in *A*–*C* and *E* represents the means ± s.e.m from 3 independent experiments. *: *p*<0.05; **: *p*<0.01; *NS*: *p*>0.05.

Since mechanosensing of matrix rigidity involves cytoskeleton contractility in mESCs and other multipotent adult stem cells, we decided to investigate whether there would be a correlation between stemness of single hESCs and their cytoskeleton contractility, by examining simultaneously expressions of Oct4 (a nuclear transcription factor and hallmark of stemness) in hESCs and their traction forces (Fig. 1). Undifferentiated hESCs, confirmed using flow cytometry analysis of SSEA-3 positive cells with a purity of 95.90%, were seeded as single cells in the complete culture medium containing basic fibroblast growth factor (bFGF) on the PDMS micropost array with the post diameter *D* of 1.83 µm, the height *L* of 12.9 µm, and the effective modulus *E_eff_* of 1.92 kPa. Live-cell traction forces as well as the percentage of Oct4 positive (Oct^+^) cells, defined as the ratio of Oct^+^ cells to the total cell number, were quantified 24 hrs after plating (Fig. 1 and Fig. 2A). It was noticeable that within 24 hrs on the PDMS micropost array, 25.6%±3.8% of single hESCs lost expression of Oct4, suggesting spontaneous differentiation. A similar level of spontaneous differentiation (16.1%±2.0%) was also observed for single hESCs plated on control vitronectin-coated tissue culture plates (with the *Young*'s modulus *E* of 10^6^ kPa) (Fig. S1). Quantification of live-cell traction forces of hESCs on the PDMS microposts showed strong positive correlations between total traction force per cell and cell spread area for both undifferentiated (Oct^+^) and differentiated (Oct^−^) hESCs (Fig. S2), similar to our previous observations with adult stem cells [7]. This observation suggested that total traction force per cell normalized by cell spread area could be used as a suitable indicator to gauge the mechanical state of single hESCs. The evaluation of cytoskeleton contractility indicated a significant difference between Oct^+^ and Oct^−^ cells, as Oct^+^ hESCs showed significantly less total traction force per cell (Fig. 1*B*) and total traction force per cell area (Fig. 1*C*) as compared to Oct^−^ cells. Vinculin, a FA protein, was used to examine the functional role of FAs in regulating the mechanosensitivity of hESCs (Fig. 1*D*). Vinculin-expressing FAs appeared to be concentrated on the cell periphery of Oct^+^ hESCs, while for Oct^−^ cells FAs were randomly distributed across the cell spreading area. Taken together, our results in Fig. 1 suggested that cytoskeleton contractility and FA formation might be intrinsic mechanical properties of hESCs correlating closely with their pluripotent state.

Next we investigated whether single hESCs could sense and respond to changes in matrix mechanics using PDMS micropost arrays with different rigidities, and whether matrix rigidity could influence pluripotency of hESCs. Here we used a modified culture condition to challenge the self-renewal of hESCs by removing bFGF from the complete culture medium. Undifferentiated hESCs were first seeded in complete medium onto three different PDMS micropost arrays with the same post diameter *D* of 1.83 µm but different post heights, whose effective modulus *E_eff_* were 1.92 kPa (soft), 14.22 kPa (medium rigid) and 1,218.4 kPa (rigid), respectively. Twenty-four hours after seeding the complete culture medium was replaced with the bFGF-free medium, and hESCs were cultured for another 24 hrs before analyzing their Oct4 expressions. Under the bFGF-free condition, we observed a significant decrease of the percentage of Oct^+^ cells as compared to the full medium condition. However, matrix rigidity appeared to play a significant role in regulating pluripotency of hESCs, as 39.5%±5.5% of single hESCs on the rigid PDMS micropost array remained as undifferentiated Oct^+^ cells, while on the medium rigid and soft PDMS micropost arrays, only 11.6%±4.8% and 2.8%±2.6% of hESCs were Oct^+^, respectively (Fig. 2*A*). To investigate whether matrix mechanics-mediated Oct4 expression in single hESCs was correlated with endogenous cytoskeleton contractility, cellular traction forces of both Oct^+^ and Oct^−^ cells were measured using PDMS micropost arrays with an *E_eff_* ranging from 1.92 to 11.54 kPa. As shown in Fig. 2*B&C*, Oct^−^ cells consistently showed greater cytoskeleton contractility than Oct^+^ cells, regardless the micropost rigidity *E_eff_*. Furthermore, both Oct^+^ and Oct^−^ cells increased their cytoskeleton contractility with *E_eff_*. Together, our results in Fig. 2 suggested that hESCs are mechanosensitive and rigid substrates are supportive for pluripotency maintenance of single hESCs. Our data also showed that increasing matrix rigidity might lead to stronger cytoskeleton contractility in hESCs as reflected by their greater traction forces measured by the PDMS micropost arrays [18].

Recent studies have indicated a functional role of nonmuscle myosin II activity in regulating the fate decision of hESCs [19], [20]. Mechanosensing of matrix rigidity by adherent mammalian cells also involves actomyosin-mediated cytoskeleton contractility [6], [21]. Thus, we decided to examine the functional role of nonmuscle myosin II activity on rigidity-dependent self-renewal of hESCs. hESCs were seeded as single cells on the PDMS micropost arrays of different rigidities at 3,000 cells/cm^2^ in the complete cell medium. Twenty four hours after cell seeding, the complete culture medium was replaced with the bFGF-free medium supplemented with 10 µM blebbistatin, a small molecule that inhibits specifically nonmuscle myosin II activity. Blebbistatin treatment for 24 hrs had no significant effect on pluripotency maintenance of hESCs on the rigid PDMS micropost array (Fig. 2*D&E*). However, a prolonged 4-day treatment of hESCs with blebbistatin resulted in loosely connected single hESCs and a significant decrease in cell density and the percentage of Oct^+^ cells (Fig. 2*D&E*). No compact and sharp edged colonies could be observed for blebbistatin-treated cells, in distinct contrast to compact colonies formed by untreated controls. Our observations in Fig. 2*D&E* were consistent with recent studies reporting that the effect of blebbistatin treatment on hESC self-renewal might be time-dependent, and long-term exposure to blebbistatin could inhibit cell division and thus negatively impact hESC self-renewal [19], [20].

Blebbistatin-treated single hESCs on the rigid PDMS micropost array appeared to behave differently from untreated cells on the soft micropost array, under both 24-hr and 4-day treatment conditions with blebbistatin (Fig. 2*E*). This observation suggested that although it is known that actomyosin activity can be down-regulated for mechanosensitive adherent cells plated on soft substrates, other molecular mechanisms might also exist and work in parallel to transduce the rigidity signal to regulate stem cell fate, such as integrin-mediated adhesion signaling [21], [22].

**Figure 3:**
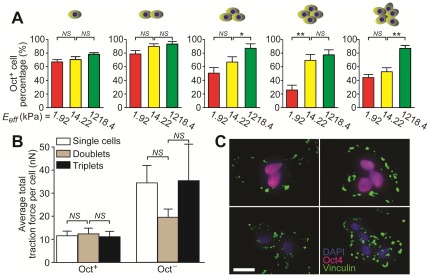
Matrix mechanics-mediated cellular functions of small aggregates of hESCs on PDMS micropost arrays with different rigidities. (*A*) Bar plots of percentage of Oct^+^ cells for clustered hESCs of different sizes as a function of the PDMS micropost rigidity. (*B*) Plot of average total traction force per cell for both Oct^+^ and Oct^−^ cells contained in different sized hESC aggregates. Data in *A* & *B* represents the means ± s.e.m from 3 independent experiments. *: *p*<0.05; **: *p*<0.01; *NS*: *p*>0.05. (*C*) Immunofluorescence images showing FA distributions in Oct^+^ (top) and Oct^−^ (bottom) hESC aggregates, as indicated by vinculin staining. Scale bar, 25 µm.

We further investigated mechanosensitivity of small aggregates of hESCs (containing *n* = 2–5 cells). hESCs were seeded at a high density (8,000 cells/cm^2^) on different PDMS micropost arrays with *E_eff_* ranging from 1.92–1,218.4 kPa in the complete culture medium. Live-cell traction force measurements were performed 24 hrs after cell seeding (Fig. S3), and percentages of Oct^+^ cells were quantified on different PDMS micropost arrays (Fig. 3*A*). Under this condition, single cells and doublets did not show significant differences on different PDMS micropost arrays with regard to their Oct4 expressions. However, it appeared that larger aggregates of hESCs (*n*>2 cells) had a greater tendency to differentiate on softer micropost arrays while maintaining their Oct4 expressions on more rigid ones (Fig. 3*A*). Our live-cell traction force results in Fig. 3*B* also showed that the average total traction force per cell for hESCs plated on the same PDMS micropost array (with *E_eff_* = 1.92 kPa) was not significantly different among single cells, doublets or triplets, for both Oct^+^ and Oct^−^ cells, suggesting that cell-cell contact might play an important role in regulating matrix mechanics-mediated pluripotency maintenance of hESCs. To examine the FA distribution in small aggregates of hESCs, immunostaining of vinculin was performed (Fig. 3*C*). Our staining images showed that similar to results for single hESCs in Fig. 1*D*, vinculin-expressing FAs were concentrated on the cell periphery of clustered Oct^+^ hESCs, while for clustered Oct^−^ cells, FAs were randomly distributed across the cell spreading area.

It was interesting to observe that there was a lack of response of single hESCs to rigidity changes in Fig. 3*A*, which might be due to the fact that bFGF in the complete culture medium might have activated mechanotransductive signaling cascades downstream of rigidity sensing of single hESCs. Future studies might be necessary to elucidate the convergence and cross-talk of different well-conserved soluble factor-mediated signal transduction pathways and the cellular mechanosensing and mechanotransduction processes to activate the elaborate intracellular signaling network in an integrated and interacting manner to regulate hESC fate.

**Figure 4:**
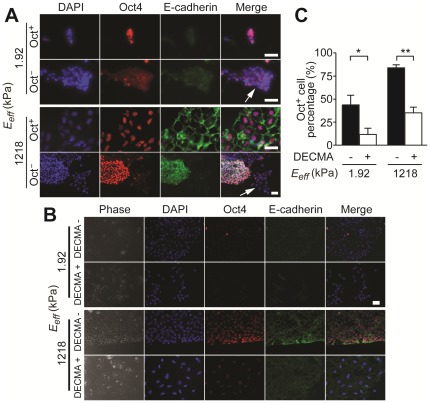
E-cadherin expression of hESCs modulated by substrate rigidity. (*A*) Immunofluorescence images taken for undifferentiated (Oct^+^) and differentiated (Oct^−^) hESC colonies on soft (*E_eff_* = 1.92 kPa) and rigid (*E_eff_* = 1,218.4 kPa) PDMS micropost arrays, as indicated. Differentiated hESC colonies were marked with an arrow. Scale bars, 50 µm. (*B*) Phase contrast and immunofluorescence images of hESCs treated with or without DECMA-1 on both soft (*E_eff_* = 1.92 kPa) and rigid (*E_eff_* = 1,218.4 kPa) PDMS micropost arrays. Scale bar, 50 µm. (*C*) Bar plot of percentage of Oct^+^ cells for DECMA-1 treated hESCs and untreated controls as a function of the PDMS micropost rigidity. Data represents the means ± s.e.m from 3 independent experiments. *: *p*<0.05; **: *p*<0.01.

Recent studies have shown that actomyosin-mediated cytoskeleton contractility and E-cadherin expression form a positive feedback loop to promote maintenance of pluripotency of hESCs [20]. Thus, in hESC aggregates with cell-cell contacts, E-cadherin expression might be correlated with CSK contractility, expression of Oct4 and pluripotency of hESCs. To examine this possibility, single hESCs were plated at 8,000 cells/cm^2^ in the complete medium on the soft (*E_eff_* = 1.92 kPa) and rigid (*E_eff_* = 1,218.4 kPa) PDMS micropost arrays. Twenty-four hours after seeding, cells were co-stained for E-cadherin and Oct4. Co-expressions of Oct4 and E-cadherin were detected in Oct^+^ hESCs on the rigid micropost array, while E-cadherin was not detectable in Oct^−^ cells on the rigid micropost array (Fig. 4*A*). In comparison, hESCs plated on the soft micropost array expressed very low levels of E-cadherin, regardless of their Oct4 expressions (Fig. 4*A*).

To examine the functional role of E-cadherin in mediating rigidity-dependent self-renewal of hESCs, we performed E-cadherin inhibition assays for hESCs cultured on both the soft (*E_eff_* = 1.92 kPa) and rigid (*E_eff_* = 1,218.4 kPa) PDMS micropost arrays. hESCs were treated with or without DECMA-1, an antibody that blocks E-cadherin activity [20], for 5 days before cells were fixed and stained for E-cadherin and Oct4. Blocking E-cadherin activity in hESCs on both the rigid and soft micropost arrays by DECMA-1 resulted in loosely connected single cells as compared to compact and sharp edged colonies for untreated controls (Fig. 4*B*). Percentages of Oct^+^ cells also decreased significantly for DECMA-1 treated hESCs as compared to untreated controls, regardless the micropost rigidity *E_eff_* (Fig. 4*C*). Together, our findings in Fig. 4 supported a possible involvement of E-cadherin in rigidity-dependent self-renewal of hESCs.

In conclusion, our results demonstrated that cytoskeleton contractility, FA formation, and E-cadherin expression were all critically involved in mechanoresponsive differentiation of hESCs. Specifically, hESCs were mechanosensitive and increased their cytoskeleton contractility with matrix rigidity, and rigid substrates were supportive for maintenance of pluripotency of hESCs. Matrix mechanics-mediated cytoskeleton contractility might be functionally correlated to E-cadherin expressions in cell-cell contacts and involved in fate decisions of hESCs. Our results highlighted the important functional link between matrix rigidity, cellular mechanics, and pluripotency of hESCs. In addition, our micropost array system provided a novel approach to characterize and understand mechanotransduction and its involvement in hESC function. Mechanotransduction in hESCs is a largely unexplored area, and advancing understanding of mechanoresponsive behaviors of hESCs will help in the design of biologically inspired *in vitro* cellular microenvironments to guide growth, differentiation, and functional assembly of hESCs.

## Materials and Methods

### Fabrication and surface functionalization of the PDMS micropost array

The PDMS micropost arrays were fabricated using the protocol described previously [23]. Briefly, silicon micropost array masters were fabricated by standard photolithography and deep reactive ion etching (DRIE). The height of the silicon micropost was modulated by controlling the DRIE etch time. The silicon masters were silanized with (tridecafluoro-1,1,2,2,-tetrahydrooctyl)-1-trichlorosilane (United Chemical Technologies, Bristol, PA) for 4 hr under vacuum. Then, 1∶10 (w/w, curing agent:base monomer) ratio PDMS prepolymer (Sylgard 184, Dow-Corning, Midland, MI) was poured over the silicon micropost master and cured at 110°C for 20 min. The negative PDMS mold was then generated by peeling off from the silicon master, oxidized with the oxygen plasma for 1 min (200 mTorr; Plasma Prep II, West Chester, PA), and silanization with (tridecafluoro-1,1,2,2,-tetrahydrooctyl)-1-trichlorosilane for 24 hrs to obtain the PDMS negative mold. To generate the final PDMS micropost array, 1∶10 ratio PDMS prepolymer was poured over the negative PDMS mold and degassed under vacuum for 10 min. Then a clean 25 cm×25 cm cover glass was placed on top of the negative mold and the whole assembly was cured at 110°C for 40 hrs. The negative mold was then peeled off to release the final PDMS micropost array. The collapsed PDMS microposts during peeling off was rescued by sonication in 100% ethanol for 30 sec followed by dry-release with liquid CO_2_ using a critical point dryer (Samdri®-PVT-3D, Tousimis, Rockville, MD).

The top surface of the PDMS micropost array was functionalized with human recombinant vitronectin (R&D system, Minneapolis, MN) to promote adhesion of hESCs. Briefly, a flat 1∶30 PDMS stamp was soaked with vitronectin at a concentration of 20 µg mL^−1^ in distilled water for 1 hr at room temperature. The PDMS stamp was then thoroughly rinsed with distilled water and blown dry with a stream of nitrogen. In parallel, the PDMS micropost array was treated with ozone generated by a UV-ozone cleaner (Jelight, Irvine, CA) for 7 min to activate the surface of the PDMS micropost array, so that the hydroxyl group generated during this process on the PDMS surface could covalently bond to vitronectin. The PDMS stamp was then placed in conformal contact with the PDMS micropost array for about 10 sec. To prevent non-specific protein absorption to the non-functionalized surface of the PDMS micropost array, the PDMS micropost array was soaked in pluronics F127 NF dissolved in PBS (0.2%, w/v; BASF, Ludwigshafen, Germany) for 30 min. For the PDMS micropost array used for traction force measurement, an additional labeling step was performed by soaking the PDMS micropost array with 1,1′-dioctadecyl-3,3,3′,3′-tetramethylindodicarbocyanine perchlorate (‘DiD’ oil; Invitrogen, Carlsbad, CA) before the treatment with the pluronics F 127 NF.

### Cell culture and seeding cells on the PDMS micropost array

hESCs (H9 and H1, obtained from WiCell, Madison, WI) were cultured on a feeder-free synthetic polymer coating (PMEDSAH) [17], [24] with the Human-Cell-Conditioned Medium (GlobalStem, Rockville, MD) supplemented with 8 ng/mL of human recombinant basic fibroblast growth factor (bFGF; Globalstem). Before plating cells, differentiated cells were removed manually by a modified pasteur pipette under a stereomicroscope (Leica MZ9.5, Leica Microsystems Inc., Buffalo Grove, IL). Then, undifferentiated colonies were collected as small cell aggregates using the STEMPRO EZPassage Disposable Stem Cell Passaging Tool (Invitrogen) in a 1.5 mL centrifuge tube. After centrifugation and a brief washing with PBS, the cell aggregates were treated with 0.5 mL 0.25% Trypsin-EDTA for 1 min. 1 mL 10% fetal bovine serum (FBS, Invitrogen) was used to stop trypsinization and was followed by an immediate centrifugation. The cell pellet was then dispersed in the StemPro serum free medium (Invitrogen) supplied with 8 ng/mL bFGF and Y27632 (a ROCK inhibitor) at 10 µM and passed through a cell strainer with the 40 µm nylon mesh (BD Biosciences, Bedford, MA) to remove large cell aggregates. The obtained single hESCs were counted and then seeded on the PDMS micropost array at a desired density.

The H1 hESC line (H1, obtained from WiCell) was also tested in this work. These hESCs were cultured on mitotically inactive mouse embryonic fibroblasts (MEFs, obtained from GlobalStem, Rockville, MD) with the standard hESC culture medium containing the knock-out serum replacement (Invitrogen), non-essential amino acid (Invitrogen), and bFGF (Invitrogen). Before plating the cells, differentiated cells were removed manually by a modified pasteur pipette under a stereomicroscope (Leica), and undifferentiated colonies were collected as small cell aggregates using the STEMPRO EZPassage Disposable Stem Cell Passaging Tool (Invitrogen). The collected cells were briefly rinsed with PBS and treated with TrypLE Select (Invitrogen) for 3 min to release the MEFs. The cells were again rinsed with PBS and then all the cells including hESCs and remaining MEFs were scraped using a cell scraper (BD Biosciences). To further remove the remaining MEFs, the solution containing the cells was transferred into a 35 mm culture dish (BD Biosciences) coated with gelatin and incubated for 45 min. Most MEFs would attach to the dish while hESCs were still in the supernatant. The supernatant was collected and centrifuged and the cell pellet was treated with 0.25% Trypsin-EDTA for 1 min. After this, the same procedure used for H9 cells was followed to obtain and seed single hESCs on the PDMS micropost array.

### Quantification of traction forces using the PDMS micropost array

For traction force measurements, the cover glass holding the PDMS micropost array was attached to a 35 mm dish with a 20 mm hole at the center (Glass-bottom-dishes, MatTek, Ashland, MA). Live-cell images were obtained using a 40× objective (1.3 NA, oil immersion; EC Plan NEOFLUAR; Carl Zeiss MicroImaging, Thornwood, NY) on a Zeiss Observer.Z1 microscope equipped with a thermoelectrically-cooled monochrome CCD camera (AxioCam HRM, Carl Zeiss MicroImaging). The microscope was further equipped with an environmental chamber to maintain the experimental environment at 37°C. A microscope stage incubator (Carl Zeiss MicroImaging) was also used to maintain 5% CO_2_. The positions of single hESCs were recorded by the AxioVision software (Carl Zeiss MicroImaging). A custom-developed MATLAB program (details of the program was described in Ref. [23]; The MathWorks, Natick, MA) was used to calculate deflection of the PDMS micropost centroid from its unbent, unloaded position, which was then converted to the horizontal traction force by multiplying with the nominal spring constant *K* of the PDMS micropost.

### Immunofluorescence staining

Cells were fixed with 4% paraformaldehyde (Electron Microscopy Sciences, Hatfield, PA) for 20 min at room temperature, and then permeabilized with 0.1% Triton X-100 (Roche Applied Science, Indianapolis, IN) for 20 min. To assay Oct4 expression, Oct4 rabbit polyclonal IgG (Santa Cruz Biotechnology, Santa Cruz, CA) primary antibody, which did not cross-react with Oct4 isoform B, was used and detected by the goat-anti-rabbit Alexa-546 secondary antibody (Invitrogen). To examine the expression of E-cadherin, mouse anti-E-Cadherin primary antibody (Invitrogen) was used and detected by the goat-anti-mouse Alexa-647 secondary antibody (Invitrogen). To examine the effect of myosin inhibition, mouse anti-nonmuscle myosin IIA primary antibody (Abcam, Cambridge, MA) was used and detected by the goat-anti-mouse Alexa-488 secondary antibody (Invitrogen). For vinculin staining, cells were incubated in an ice-cold cytoskeleton buffer (50 mM NaCl, 150 mM sucrose, 3 mM MgCl_2_, 1 µg/mL aprotinin, 1 µg/mL leupeptin, 1 µg/mL pepstatin, and 2 mM PMSF) for 1 min, followed by 1 min in the cytoskeleton buffer supplemented with 0.5% Triton X-100. After that, the mouse anti-vinculin primary antibody (Sigma-Aldrich, St. Louis, MO) was used and detected by the goat-anti mouse Alexa-488 secondary antibody.

### Inhibition experiments

Rat monoclonal antibody against E-cadherin (DECMA-1) (Abcam, Cambridge, MA) was added to media at 12 µg/mL. Blebbistatin (Sigma) was added to media at 10 µM. Media containing DECMA-1 or blebbistatin was refreshed every day.

### Statistics

For comparisons between two data sets, *p* value was calculated using the student *t*-test function in Excel (Microsoft, Seattle, WA). For comparisons between multiple data sets, *p* value was calculated using one way ANOVA analysis followed by the Tukey post hoc test.

## Supporting Information

Figure S1 Spontaneous differentiation of single hESCs plated on tissue culture plates coated with vitronectin at different densities. (*A*) Immunofluorescence images showing single hESCs plated at different densities stained for DAPI and Oct4. Scale bar, 200 µm. (*B*) Bar plot of percentages of Oct^+^ cells for single hESCs plated on the tissue culture plates as a function of initial cell seeding density.
(TIF)

Figure S2 Correlative analysis of cell morphology and traction force for single Oct^+^ (left) and Oct^−^ (right) hESCs during rigidity-sensing. Total traction force per single hESCs was plotted against hESC spread area. Each data point represents an individual cell. Data were collected from three different PDMS micropost arrays (Top row, *A&B*: *E_eff_* = 11.54 kPa; Middle row, *C&D*: *E_eff_* = 7.22 kPa; Bottom row, *E&F*: *E_eff_* = 1.92 kPa). Data trends were compared with the linear least square fitting (red lines, with the slope and *R^2^* values indicated).
(TIF)

Figure S3 Representative fluorescence images showing measurements of traction forces for small aggregates of Oct^+^ and Oct^−^ hESCs plated on the PDMS micropost array.
(TIF)
