# Supplementary figures and images for: Mechanics Regulates Fate Decisions of Human Embryonic Stem Cells
Source: PLoS One. 2012 May 16;7(5):e37178. doi: 10.1371/journal.pone.0037178 (PMC3353896; doi:10.1371/journal.pone.0037178)

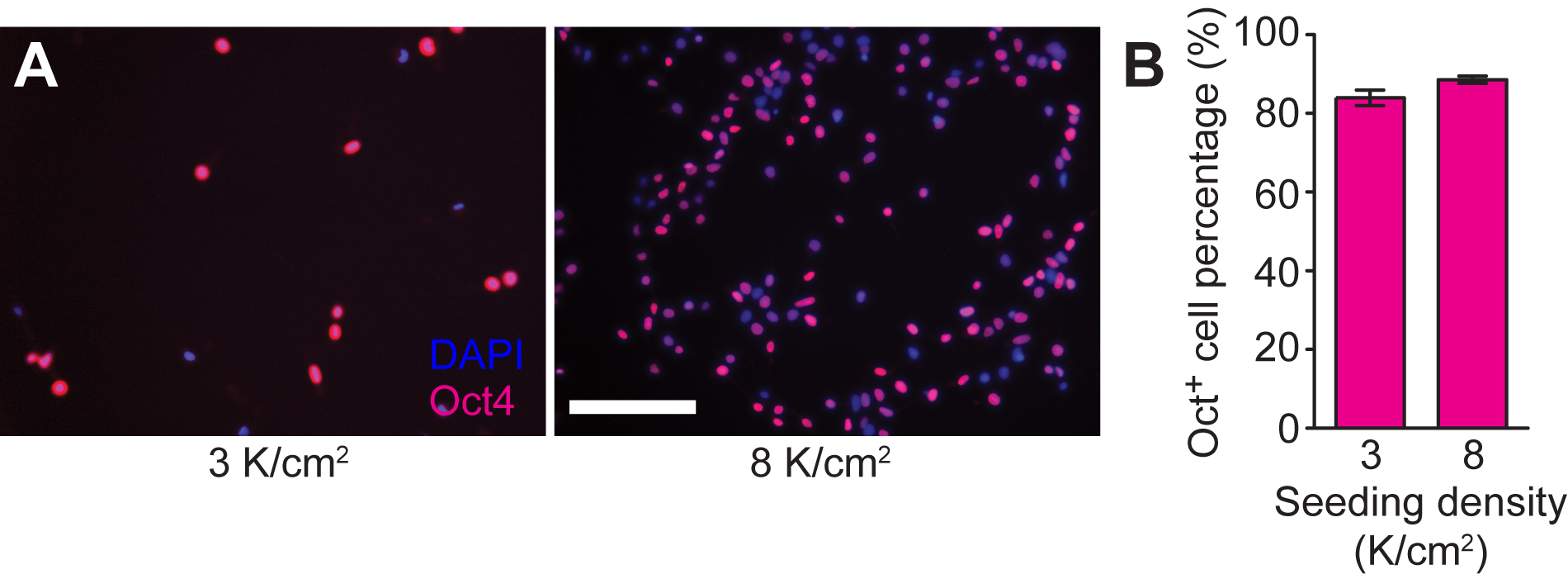

Supplement: Figure S1 — Spontaneous differentiation of single hESCs plated on tissue culture plates coated with vitronectin at different densities. (A) Immunofluorescence images showing single hESCs plated at different densities stained for DAPI and Oct4. Scale bar, 200 µm. (B) Bar plot of percentages of Oct+ cells for single hESCs plated on the tissue culture plates as a function of initial cell seeding density. (TIF) [file pone.0037178.s001.tif]

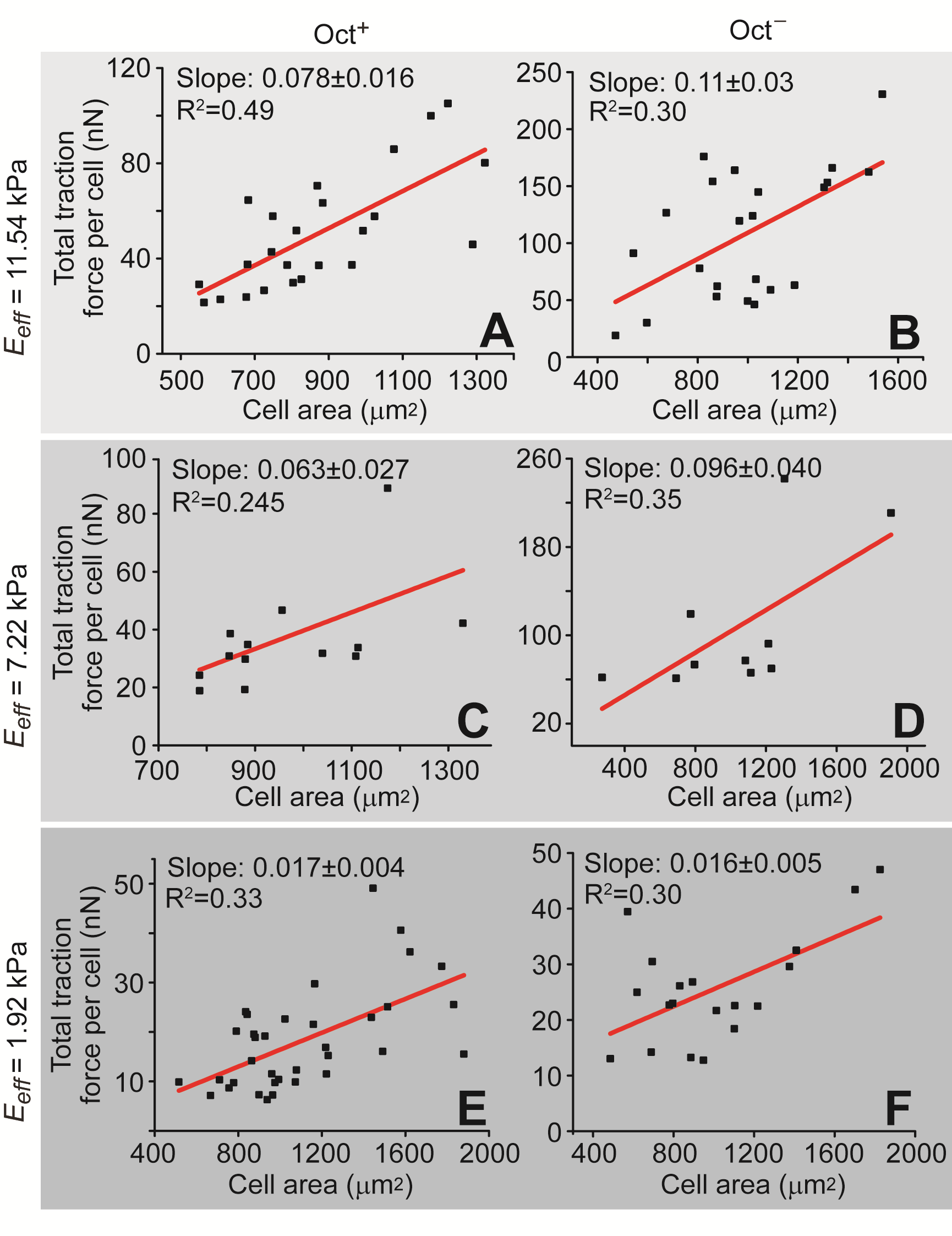

Supplement: Figure S2 — Correlative analysis of cell morphology and traction force for single Oct+ (left) and Oct− (right) hESCs during rigidity-sensing. Total traction force per single hESCs was plotted against hESC spread area. Each data point represents an individual cell. Data were collected from three different PDMS micropost arrays (Top row, A&B: Eeff = 11.54 kPa; Middle row, C&D: Eeff = 7.22 kPa; Bottom row, E&F: Eeff = 1.92 kPa). Data trends were compared with the linear least square fitting (red lines, with the slope and R2 values indicated). (TIF) [file pone.0037178.s002.tif]

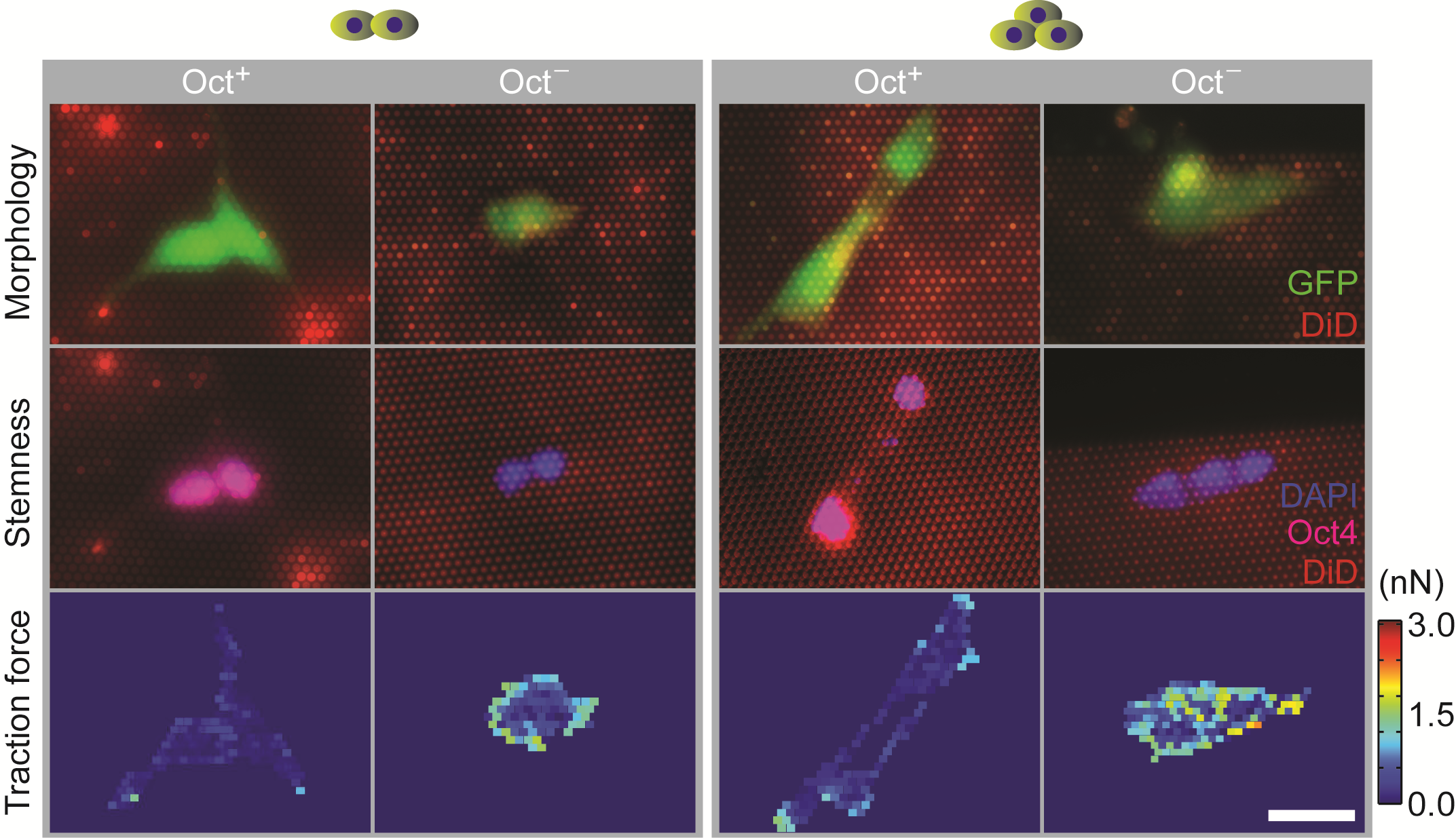

Supplement: Figure S3 — Representative fluorescence images showing measurements of traction forces for small aggregates of Oct+ and Oct− hESCs plated on the PDMS micropost array. (TIF) [file pone.0037178.s003.tif]
